# Effect of extracorporeal liver support by MARS and Prometheus on serum cytokines in acute-on-chronic liver failure

**DOI:** 10.1186/cc5119

**Published:** 2006-12-07

**Authors:** Vanessa Stadlbauer, Peter Krisper, Reingard Aigner, Bernd Haditsch, Aleksandra Jung, Carolin Lackner, Rudolf E Stauber

**Affiliations:** 1 Department of Internal Medicine, Division of Gastroenterology and Hepatology, Medical University of Graz, Auenbruggerplatz 15, 8036 Graz, Austria; 2 Department of Internal Medicine, Division of Nephrology and Hemodialysis, Medical University of Graz, Auenbruggerplatz 15, 8036 Graz, Austria; 3 Department of Radiology, Division of Nuclear Medicine, Medical University of Graz, Auenbruggerplatz 9, 8036 Graz, Austria; 4 Department of Medical Physics, AGH University of Science and Technology, Mickiewicza Ave, PL-30 059 Krakow, Poland; 5 Institute of Pathology, Medical University of Graz, Auenbruggerplatz 25, 8036 Graz, Austria

## Abstract

**Introduction:**

Cytokines are believed to play an important role in acute-on-chronic liver failure (ACLF). Extracorporeal liver support systems may exert beneficial effects in ACLF via removal of cytokines. At present, two systems are commercially available, the Molecular Adsorbent Recirculating System (MARS™) and Fractionated Plasma Separation, Adsorption and Dialysis (Prometheus™). The aim of this study was to compare the effects of MARS and Prometheus treatments on serum cytokine levels and their clearances.

**Methods:**

Eight patients with ACLF underwent alternating treatments with either MARS or Prometheus in a randomized cross-over design. Thirty-four treatments (17 MARS, 17 Prometheus) were available for analysis. Serum cytokines were measured before and after each treatment, and cytokine clearance was calculated from paired arterial and venous samples and effective plasma flow one hour after the start of treatment.

**Results:**

Baseline serum levels of interleukin (IL)-6, IL-8, IL-10, tumor necrosis factor-alpha (TNF-α), and soluble TNF-α receptor 1 were significantly elevated in patients with ACLF. Measurable plasma clearances were detected for all cytokines tested, but no significant changes in serum levels of any cytokine were found after treatments with MARS or Prometheus. In MARS treatments, IL-10 was cleared from plasma more efficiently than IL-6. Clearance of IL-10 was higher in Prometheus than in MARS treatments.

**Conclusion:**

Cytokines are cleared from plasma by both MARS and Prometheus, but neither system is able to change serum cytokine levels. This discrepancy is probably due to a high rate of cytokine production in patients with ACLF.

## Introduction

Acute-on-chronic liver failure (ACLF) has been defined as acute deterioration of liver function in cirrhotic patients over a period of two to four weeks, usually precipitated by gastrointestinal bleeding, infection, binge drinking, or surgery, and is associated with progressive jaundice, hepatic encephalopathy and/or hepatorenal syndrome, and signs of multi-organ dysfunction [1]. ACLF has been shown to carry poor prognosis, with an in-hospital mortality ranging from 50% to 66% [2,3]. Several extracorporeal liver support systems have been developed to improve prognosis in acute liver failure as well as ACLF, and in a recent meta-analysis, artificial liver support was shown to reduce mortality in ACLF as compared with standard medical treatment [4]. Recently, research has focused on cell-free systems, such as the Molecular Adsorbent Recirculating System (MARS™; Gambro AB, Stockholm, Sweden) and the Fractionated Plasma Separation, Adsorption and Dialysis system (Prometheus™; Fresenius Medical Care AG & Co. KGaA, Homburg, Germany), which provide elimination of albumin-bound toxins and thus are believed to enhance liver regeneration [5,6].

The designs of MARS and Prometheus differ considerably. In MARS, blood is dialyzed across an albumin-impermeable membrane with a molecular weight cutoff of 60 kDa against 20% human serum albumin, which is continuously cleansed by subsequent passage through columns of charcoal and an anion exchange resin. Water-soluble substances such as NH_4_^+ ^are removed by a low-flux dialyzer connected to the secondary circuit [6,7]. In Prometheus, the patient's plasma, containing the albumin, is separated by a membrane with a molecular weight cutoff of approximately 250 kDa and directly passed over two columns containing different adsorbents. Water-soluble substances are cleared by a high-flux dialyzer directly inserted into the blood circuit [5,8-10].

Whereas elimination of albumin-bound substances such as bilirubin or bile acids has been well defined for both MARS and Prometheus [8,11-16], little is known of their impact on pathophysiology of liver failure. Beneficial effects of extracorporeal liver support might be evoked by modifying the patient's response to the disease. Systemic inflammatory reaction, characterized by a predominantly proinflammatory cytokine profile, may cause the transition from stable cirrhosis to ACLF [17]. Proinflammatory cytokines are believed to mediate hepatic inflammation, apoptosis and necrosis of liver cells, cholestasis, and fibrosis [18]. Therefore, it has been hypothesized that removal of proinflammatory cytokines could be beneficial in patients with ACLF [19]. However, data on the effect of extracorporeal liver assist devices on serum cytokine levels are controversial [19-26]. This might be attributed to the inhomogeneous patient groups studied and to the diversity of tests used by different groups.

In previous studies performed in the same group of patients, we compared the elimination capacity of both systems for bilirubin fractions and bile acids and found superior removal of bilirubin (especially of the unconjugated fraction) by Prometheus but similar removal of bile acids by both systems [12,15]. The aim of the present study was to evaluate and compare the removal of several cytokines associated with inflammatory liver disease by MARS and Prometheus in patients with ACLF.

## Materials and methods

### Patients

Seven consecutive patients with ACLF as defined above and one patient with primary dysfunction of a liver graft after transplantation for decompensated liver cirrhosis were enrolled. In four of the eight patients, alcoholic hepatitis was the precipitating event causing acute decompensation of preexisting alcoholic liver cirrhosis (Table 1). To assess severity of liver disease, Child-Pugh score and model for end-stage liver disease (MELD) score were calculated at baseline [27,28]. The acute physiology and chronic health evaluation II (APACHE II) and sepsis-related organ failure assessment (SOFA) scores were used to estimate multi-organ dysfunction [29,30]. Besides, the presence or absence of a systemic inflammatory response syndrome (SIRS) was documented [31].

Patients were allocated to MARS or Prometheus treatments in a randomized cross-over design. By means of sealed envelopes, patients were randomly assigned to start with either MARS or Prometheus and underwent alternating MARS and Prometheus treatments on two to eight consecutive days. The number of treatments applied was dependent on the clinical course. The study protocol was approved by the Ethics Committee of the Medical University of Graz, and informed consent was obtained in accordance with the Declaration of Helsinki.

### Extracorporeal liver support

MARS and Prometheus treatments were performed for six hours at identical blood and dialyzate flows in all patients (200 and 300 ml/minute, respectively), and the same dialysis machine (4008 H; Fresenius Medical Care AG & Co. KGaA) was used during the entire study. The flows in the secondary circuit were set to 200 ml/minute in MARS and 300 ml/minute in Prometheus as recommended by the manufacturers. The dialyzate contained glucose (1 g/l) and magnesium (0.75 mmol/l), and sodium, potassium, and bicarbonate were adjusted to fit each patient's needs. Heparin, epoprostenol (Flolan^®^; GlaxoSmithKline, Vienna, Austria) (4 ng/kg per minute), or both were used for anticoagulation, and activated partial thromboplastin time was aimed to remain less than 100 seconds. All patients were treated via a central venous catheter. Infusions of albumin or packed red cells were not allowed during treatments.

### Liver biopsy

In four of the eight patients, a transjugular liver biopsy was performed prior to extracorporeal liver support. Liver biopsy specimens were routinely stained with hematoxylin and eosin and chromotrope aniline blue and were assessed for the presence of steatohepatitis by one of us (CL).

### Cytokine assays

Blood samples were drawn at baseline, at the end of, and one hour after the end of each treatment. In addition, paired samples were obtained from the afferent and efferent branches of the central venous line at one hour. Serum samples from 28 voluntary blood donors were used as controls. Samples were centrifuged after 30 minutes, and serum aliquots were stored frozen at -70°C for later assay of cytokines. Interleukin (IL)-6 was analyzed by a chemiluminescent assay (Immulite 2000; DPC Biermann GmbH, Bad Nauheim, Germany). Enzyme-linked immunosorbent assay (ELISA) was used to measure the levels of IL-8, IL-10, tumor necrosis factor-alpha (TNF-α) (Quantikine^®^; R&D Systems Inc., Minneapolis, MN, USA), and soluble TNF-α receptor 1 (sTNF-αR1) (BMS203CE; Bender MedSystems GmbH, Vienna, Austria). The methods were applied according to the manufacturers' recommendations. In brief, for IL-6, a solid-phase, enzyme-labeled, chemiluminescent sequential immunometric assay was performed. A 100-μl serum sample was added to the test tube containing an assay-specific coated bead and incubated at 37°C for 30 minutes. Unbound material was washed from the bead, and chemiluminescent substrate was added. Light emission was read with a high-sensitivity photon counter. For cytokine ELISA (IL-8, IL-10, TNF-α, and sTNF-αR1), analyte-specific antibodies (capture antibodies) were pre-coated onto a microplate. A 100-μl serum sample was added, and any analyte present was bound by the immobilized antibody. An enzyme-linked analyte-specific detection antibody then was bound to a second epitope on the analyte, forming the analyte-antibody complex. Substrate was added and optical density was read on a microplate reader.

### Calculations

Because cytokines are cleared from the plasma fraction of whole blood, plasma clearance (PlCl) rather than blood clearance was chosen. PlCl was calculated at one hour of treatment from paired afferent (a) and efferent (e) samples and from plasma flow. In treatments in which excess body water has to be removed by ultrafiltration, the concentration in the venous sample may be increased due to the effects of hemoconcentration on albumin and albumin-bound solutes. Failure to account for this will lead to an underestimation of PlCl. PlCl corrected for the effect of hemoconcentration was determined as follows: PlCl = [(1 - e/a) × Qp] + [UF × (e/a)], where Qp is plasma flow (Qp = blood flow × [1 - hematocrit], in milliliters per minute) and UF is ultrafiltration rate (in milliliters per minute).

### Statistics

Results are expressed as median (Q1; Q3) unless indicated otherwise. To analyze the relationship between variables, linear regression analysis was performed. Groups were compared by the Mann-Whitney test and, in the case of paired samples, by the Wilcoxon test. When more than two groups were compared, one-way analysis of variance with Dunnett T3 *post hoc *analysis was used. A *p *value less than 0.05 was considered statistically significant.

## Results

Between March 2003 and April 2004, eight patients were enrolled and 34 treatments (17 MARS and 17 Prometheus) were available for analysis. Patient characteristics at baseline and clinical outcome are presented in Table 1. Treatments were well tolerated, and no major procedure-related adverse events occurred. Therapy had to be intermittently interrupted up to 30 minutes twice during MARS (leakage, clotting) and three times during Prometheus (clotting) but was continued afterward.

Serum levels of all cytokines were below the upper limit of normal in 28 voluntary blood donors (21 male, 7 female; age 53 ± 3 years) who served as controls. Liver histology revealed cirrhosis in all four patients who had a liver biopsy and superimposed steatohepatitis in two patients who had a history of recent heavy alcohol abuse.

**Figure 1:**
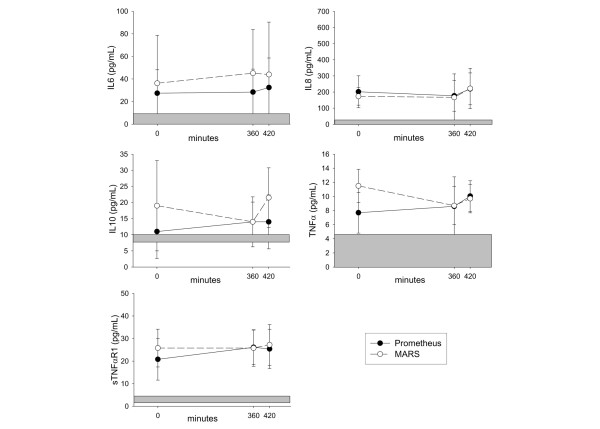
Influence of six hour treatments with MARS™ or Prometheus™ on serum cytokine levels. Levels of individual cytokines are shown at the beginning of, the end of, and 60 minutes after treatments with MARS (open circles, dashed lines) or Prometheus (filled circles, unbroken lines). Values are expressed as median (Q1; Q3). Shaded areas indicate normal range. Cytokine levels did not change significantly during treatments with MARS or Prometheus. IL, interleukin; MARS, molecular adsorbent recirculating system; sTNF-αR1, soluble tumor necrosis factor-alpha receptor 1; TNF-α, tumor necrosis factor-alpha.

**Figure 2:**
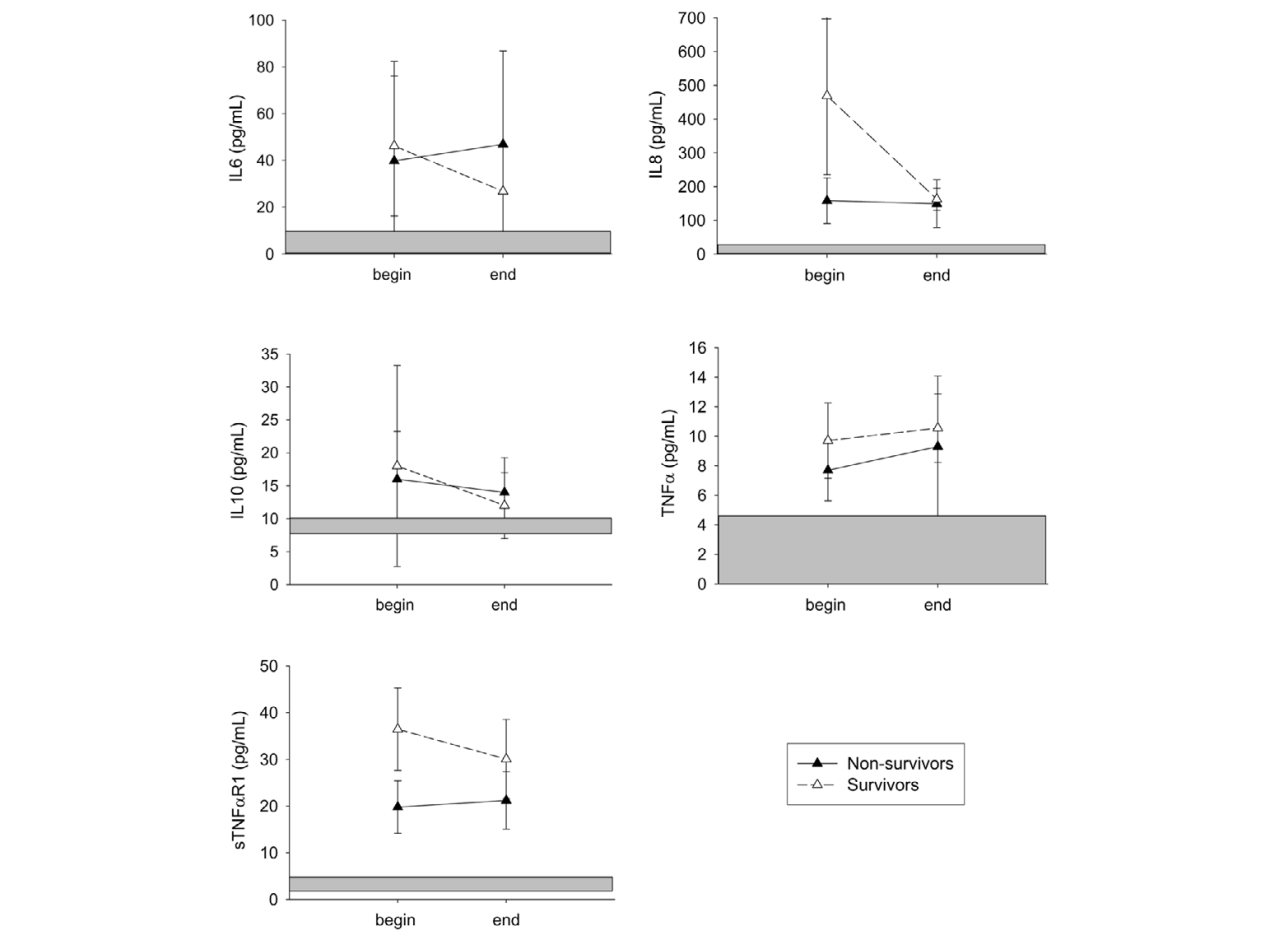
Course of serum cytokine levels during the study. Levels of individual cytokines are shown at the beginning (before first treatment) and at the end (after last treatment) of the study period, which consisted of two to eight treatments (four treatments on average; Table 1). Results are presented separately for survivors (*n *= 4; open triangles, dashed lines) and non-survivors (*n *= 4; filled triangles, unbroken lines) at day 30. Values are expressed as median (Q1; Q3). Shaded areas indicate normal range. Cytokine levels were not different between survivors and non-survivors at any time point or between the beginning and the end of the study period in both survivors and non-survivors. IL, interleukin; sTNF-αR1, soluble tumor necrosis factor-alpha receptor 1; TNF-α, tumor necrosis factor-alpha.

At baseline, serum levels of IL-6, IL-8, IL-10, TNF-α, and sTNF-αR1 were elevated in patients with ACLF as compared with controls (Figures 1 and 2). No differences in baseline levels between patients undergoing MARS or Prometheus as first treatment were noted. Likewise, no difference in baseline levels between survivors and non-survivors at day 30 after admission was found.

A measurable PlCl was found for all cytokines studied (Table 2). In MARS treatments, PlCl of IL-10 was significantly higher than that of IL-6. In Prometheus treatments, no differences in cytokine clearance were observed. Prometheus cleared IL-10 from plasma more efficiently than MARS.

No significant changes in IL-6, IL-8, IL-10, TNF-α, and sTNF-αR1 serum levels could be found in the course of six hour treatments with MARS or Prometheus, and no significant rebound 60 minutes after the treatment was noted for any of the tested cytokines (Figure 1). Cytokine levels were not different between survivors and non-survivors (day 30) at any time point, and no differences were found between the beginning and the end of the treatment series (Figure 2).

In four of the eight patients studied, alcoholic hepatitis was the precipitating event. When baseline cytokine levels of these four patients were compared with those of the non-alcoholic patients, no significant differences were found (data not shown). Likewise, baseline cytokine levels were not different between patients with (*n *= 4) or without (*n *= 4) renal failure as defined by a baseline serum creatinine level above normal (data not shown).

Baseline IL-6 values and IL-8 values tended to be higher in patients with SIRS, whereas no difference in IL-10, TNF-α, and sTNF-αR1 levels was found in patients with or without SIRS. When linear regression analysis was performed, both IL-10 (R = -0.73, *p *< 0.05; Y = 13 - [0.1 × X]) and sTNF-αR1 (R = 0.84, *p *< 0.05; Y = -136 + [13.2 × X]) were significantly related to Child-Pugh score. In contrast, none of the cytokines tested correlated with MELD, SOFA, or APACHE II score.

## Discussion

In the present study in patients with ACLF, we observed elevated serum levels of five cytokines commonly associated with inflammatory liver disease (IL-6, IL-8, IL-10, TNF-α, and sTNF-αR1) at baseline. Both MARS and Prometheus treatments showed measurable clearances for all cytokines studied. However, neither MARS nor Prometheus could lower serum levels of any cytokine.

Elevated serum levels of several cytokines, including TNF-α, sTNF-αR1, sTNF-αR2, IL-2, IL-2R, IL-4, IL-6, IL-8, IL-10, and interferon-γ, have been described in patients with ACLF [19,22], whereas other studies reported normal TNF-α levels in patients with ACLF or acute liver failure [20,22,24]. Elevated levels of circulating cytokines in ACLF may be the result of increased production due to endotoxemia, cytokine release by necrotic liver cells, and/or reduced hepatic removal. TNF-α can induce apoptosis of hepatocytes, especially in alcoholic liver disease when hepatocytes are sensitized to TNF-α-induced apoptosis [32]. Therefore, removal of proinflammatory cytokines such as TNF-α from plasma might be considered beneficial. However, cytokines such as TNF-α and IL-6 may also promote liver regeneration by inducing acute-phase proteins and hepatic proliferation and by exhibiting anti-apoptotic effects [18,32]. Because cytokines represent not only endocrine but also autocrine and paracrine effector molecules, it should be pointed out that elevated systemic levels are not representative of their role in the pathophysiology of liver failure.

Extracorporeal liver support systems use membranes with a higher molecular weight cutoff than conventional hemofilters and should therefore facilitate the elimination of larger molecules such as cytokines. Specifically, the molecular weight cutoff of the MARS membrane (60 kDa) is higher than the molecular weight of most cytokines (Table 2). However, previous studies on the removal of cytokines by MARS have produced conflicting results (Table 3). In patients with ACLF, MARS was shown to remove IL-8 over the activated charcoal column and sTNF-αR1 over the dialysis membrane, but no effect on serum levels of any cytokine tested was found [19]. This finding is consistent with a study in children with acute Wilson's disease reporting the transfer of TNF-α and IL-6 into the albumin circuit [21]. In contrast, four other studies that included patients with ACLF or acute liver failure reported a significant decrease of several cytokines in the course of MARS treatments [20,22,23,33]. Finally, in a Chinese study in patients with multi-organ dysfunction syndrome, MARS was able to lower TNF-α, IL-2, IL-6, and IL-8 [25]. Because Prometheus has a molecular weight cutoff of 250 kDa, cytokines should be readily transferred to the secondary circuit. At present, only one study on cytokine removal by Prometheus is available showing that TNF-α and IL-6 levels were not changed significantly during treatment [9].

Continuous renal replacement therapy *per se *may remove cytokines from plasma by convection and membrane adsorption (reviewed in [34]). However, removal of cytokines is not sufficient to result in a significant and sustained effect on plasma concentrations. This low efficiency has been attributed to rapid saturation of easily accessible binding sites on the membrane as well as inefficient use of less accessible binding sites due to a low convective driving force. The authors suggest that optimal mediator removal might be obtained by a combination of a high transmembrane pressure and frequent membrane changes, but this would not be feasible in clinical practice [34]. Thus, alternative devices with specific cytokine adsorbers are needed but are still at the developmental stage.

A recent study from Belgium demonstrated an improvement in mean arterial pressure and systemic vascular resistance in MARS but not Prometheus treatments, which has been attributed to a higher capacity for removal of endogenous vasoactive substances by MARS [35]. However, it cannot be ruled out that in Prometheus such vasodilators are cleared to a similar extent but possible beneficial hemodynamic effects are counterbalanced by a blood pressure drop due to the larger extracorporeal volume. Increased production of proinflammatory cytokines such as TNF-α has been suggested to represent an important mechanism for the circulatory changes observed in ACLF. Thus, removal of TNF-α by extracorporeal liver support could have beneficial hemodynamic effects. However, in the present study, we could not demonstrate any changes in serum cytokine levels during treatment sessions nor any differences between the two devices. These data are consistent with the findings by Sen and colleagues [19] for MARS and by Rifai and colleagues [9] for Prometheus.

It should be noted that comparison of data between different studies is hampered by the lack of standardization for cytokine assays. Cytokines and their soluble receptors may be measured by bioassays or immunoassays such as ELISA. The performance of ELISA methods is largely dependent on the quality of the capture antibodies used. Further potential sources of error include detection of degraded cytokines that are immunoreactive but not biologically active, matrix effects, presence of cytokine inhibitors, and inadequate sample storage [36].

Several prognostic scoring systems have been developed for patients with chronic liver disease as well as for patients admitted to an intensive care unit. In addition, serum levels of proinflammatory cytokines have been linked to the development of multi-organ failure and IL-8 was found to correlate with APACHE II score and mortality rate [37,38]. A study on 251 non-selected critically ill patients revealed APACHE III score to be the best predictor and detectable TNF-α a weak independent predictor of death [39]. We could not demonstrate correlations between serum levels of any cytokine and APACHE II or SOFA score, but interestingly, both IL-10 and sTNF-αR1 correlated with Child-Pugh score, an index of liver dysfunction.

## Conclusion

The present study demonstrates marked elevations of serum cytokine levels in patients with ACLF. Both MARS and Prometheus were able to clear cytokines from plasma, but they did not change serum cytokine levels significantly. This apparent discrepancy is probably due to a high rate of ongoing cytokine production in ACLF counterbalancing elimination within the extracorporeal circuits. These findings should temper a liberal use of current extracorporeal liver support systems in intensive care medicine and promote further research in the development of cytokine-specific adsorbents.

## Key messages

• In ACLF, serum levels of IL-6, IL-8, IL-10, TNF-α, and sTNF-αR1 are elevated. These cytokines may be involved in the pathogenesis of ACLF.

• MARS and Prometheus are cell-free extracorporeal liver support systems providing elimination of albumin-bound as well as water-soluble toxins that accumulate in liver failure.

• Both MARS and Prometheus show measurable clearance for IL-6, IL-8, IL-10, TNF-α, and sTNF-αR1.

• However, neither MARS nor Prometheus treatment is able to reduce serum levels of these cytokines, presumably due to high production rates.

• Alternative concepts are needed for effective removal of cytokines (for example, systems using cytokine-specific adsorbents).

## Abbreviations

ACLF = acute-on-chronic liver failure; APACHE II = acute physiology and chronic health evaluation II; ELISA = enzyme-linked immunosorbent assay; IL = interleukin; MARS = molecular adsorbent recirculating system; MELD = model for end-stage liver disease; PlCl = plasma clearance; SIRS = systemic inflammatory response syndrome; SOFA = sepsis-related organ failure assessment; sTNF-αR1 = soluble tumor necrosis factor-alpha receptor 1; TNF-α = tumor necrosis factor-alpha.

## Competing interests

VS, PK, and AJ received a travel grant from Fresenius Medical Care AG & Co. KGaA. All other authors declare that they have no competing interests.

## Authors' contributions

VS acquired, analyzed, and interpreted the data, drafted the manuscript, and was involved in revising the manuscript. PK made substantial contributions to the conception and design of the clinical study, carried out the extracorporeal treatments, and was involved in revising the manuscript. RA supervised the cytokine analysis and was involved in revising the manuscript. BH carried out the extracorporeal treatments, collected the serum samples, and was involved in revising the manuscript. AJ was involved in data analysis and in revising the manuscript. CL performed the histological analysis and was involved in revising the manuscript. RES made substantial contributions to the conception and design of the clinical study, selected the patients for extracorporeal liver support, analyzed and interpreted the data, and revised the manuscript. All authors read and approved the final manuscript.

## Figures and Tables

**Table 1:** Patient characteristics

Patient	Age (years)	Gender	Underlying disease	Precipitating event	Follow-up (days)	Cause of death	M	P	Creatinine (mg/dl)	INR	Bilirubin (mg/dl)	Albumin (g/dl)	MELD score^a^	Child-Pugh score	SOFA score
1	53	Female	Cirrhosis, chronic HCV	SBP	6	Sepsis	2	2	1.0	3.15	16	3.0	30	12	14
2	55	Male	Alcoholic cirrhosis	Alcoholic hepatitis	30	Sepsis	2	2	2.3	3.10	39	2.8	41	12	15
3	61	Male	Metastatic colon cancer	Liver resection	3	Multi-organ failure	1	1	0.6	1.10	25	2.8	15	11	14
4	51	Male	Alcoholic cirrhosis	Bleeding	9	Multi-organ failure	2	2	2.5	2.13	33	2.9	37	13	12
5	65	Female	Alcoholic cirrhosis	Alcoholic hepatitis	106	Sepsis	4	4	1.8	1.96	37	2.9	33	12	13
6	60	Male	Alcoholic cirrhosis	Alcoholic hepatitis	Alive, OLT day 25	n/a	2	2	5.9	2.70	38	3.0	48	13	12
7	61	Male	OLT, graft dysfunction	n/a	625	Lung cancer	2	2	1.1	1.18	20	3.3	21	9	15
8	56	Female	Alcoholic cirrhosis	Alcoholic hepatitis	Alive	n/a	2	2	1.0	2.10	26	2.4	27	12	11

^a^MELD score was obtained using the MELD calculator at the website of the Mayo Clinic (Rochester, MN, USA), . HCV, hepatitis C virus; INR, international normalized ratio; M, MARS™ (molecular adsorbent recirculating system) sessions; MELD, model for end-stage liver disease; n/a, not applicable; OLT, orthotopic liver transplantation; P, Prometheus™ sessions; SBP, spontaneous bacterial peritonitis; SOFA, sepsis-related organ failure assessment.

**Table 2:** Plasma clearance of cytokines

	Molecular weight (kDa)	PlCl at 60 minutes (ml/minute)	
		MARS™	Prometheus™
IL-6	21–28	3 (-1; 6)	4 (2; 20)
IL-8	8–10	17 (0; 28)	3 (-1; 35)
IL-10	35–40	16 (5; 26)^a^	46 (19; 60)^b^
TNF-α	17	29 (-114; 31)	25 (-5; 47)
sTNF-αR1	55	2 (-2; 9)	12 (3; 16)

^a^*p *< 0.05 versus IL-6; ^b^*p *< 0.05 versus MARS. Values (ml/minute) are expressed as median (Q1; Q3). IL, interleukin; MARS, molecular adsorbent recirculating system; PlCl, plasma clearance; sTNF-αR1 = soluble tumor necrosis factor-alpha receptor 1; TNF-α = tumor necrosis factor-alpha.

**Table 3:** Overview of published data on the effect of MARS™ and Prometheus™ on serum cytokines

Device	Reference	Patients *n*/Diagnosis	IL-6 Method/Effect	IL-8 Method/Effect	IL-10 Method/Effect	TNF Method/Effect	sTNF-αR1+2 Method/Effect
MARS	Guo *et al*. [22]	11/ALF 13/ACLF	FACS/Decrease	ELISA/Decrease	FACS/No change	FACS/Decrease	Not available
MARS	Ambrosino *et al*. [20]	17/ACLF	Not provided/Increase	Not available	Not available	Not provided/Decrease	Not available
MARS	Sen *et al*. [19]	18/ACLF	ELISA/No change	ELISA/No change	ELISA/No change	ELISA/No change	ELISA/No change
MARS	Auth *et al*. [21]	2/ALF	ELISA/Decrease in albumin circuit	Not available	Not available	ELISA/Decrease in albumin circuit	Not available
MARS	Isoniemi *et al*. [33]	49/ALF	ELISA/No change	ELISA/No change	ELISA/Decrease	ELISA/No change	Not available
MARS	Di Campli *et al*. [23]	10/ALF	ELISA/Decrease	Not available	Not available	ELISA/Decrease	Not available
Prometheus	Rifai *et al*. [9]	7/ACLF 2/Graft dysfunction	ELISA/No change	Not available	Not available	ELISA/No change	Not available

Only full papers published in English are included. ACLF, acute-on-chronic liver failure; ALF, acute liver failure; ELISA, enzyme-linked immunosorbent assay; FACS, fluorescence-activated cell sorting; IL, interleukin; MARS, molecular adsorbent recirculating system; TNF, tumor necrosis factor; TNF-R, tumor necrosis factor-receptor.
